# Cognitive load affects early processes involved in mentalizing robot behaviour

**DOI:** 10.1038/s41598-022-19213-5

**Published:** 2022-09-02

**Authors:** Nicolas Spatola, Serena Marchesi, Agnieszka Wykowska

**Affiliations:** 1 Social Cognition in Human-Robot Interaction Laboratory, Italian Institute of Technology, Genoa, Italy; 2 Artimon Perspectives, Paris, France

**Keywords:** Neuroscience, Psychology

## Abstract

How individuals interpret robots’ actions is a timely question in the context of the general approach to increase robot’s presence in human social environment in the decades to come. Facing robots, people might have a tendency to explain their actions in mentalistic terms, granting them intentions. However, how default or controllable this process is still under debate. In four experiments, we asked participants to choose between mentalistic (intentional) and mechanistic (non-intentional) descriptions to describe depicted actions of a robot in various scenarios. Our results show the primacy of mentalistic descriptions that are processed faster than mechanistic ones (experiment 1). This effect was even stronger under high vs low cognitive load when people had to decide between the two alternatives (experiment 2). Interestingly, while there was no effect of cognitive load at the later stages of the processing arguing for controllability (experiment 3), imposing cognitive load on participants at an early stage of observation resulted in a faster attribution of mentalistic properties to the robot (experiment 4). We discuss these results in the context of the idea that social cognition is a default system.

## Introduction

The future of human–robot interactions (HRI) will be framed by the way individuals will represent robots and robots’ actions. Facing a robot (especially if it has human-like appearance), humans tend to attribute to it human characteristics such as the capacity to reason or to have intentions^1^. However, the influence of the context, especially the modulation of the cognitive resources allocated, on this process remains poorly understood. In the present study, we investigated the tendency to attribute intentionality to robots’ behaviours in the context of cognitive control.

According to Dennett^2,3^, whenever individuals try to predict a physical phenomenon such as the trajectory of a kicked ball they rely on rules determined by physics. This strategy is what Dennett defines as physical stance. At a more abstract level, when systems are more complex, individuals would rely on how the system was designed to function, in doing so, they are adopting the design stance. However, more complex phenomena, such as human behaviour, are difficult to be efficiently predicted or explained using physical or design principles. To explain others’ behaviour, people tend to adopt the intentional stance. The intentional stance relies on mentalizing, and in particular on the attribution of mental states such as intentions, in order to explain behaviour. In a nutshell, Dennett’s philosophical proposal assumes a distinction between how physical phenomena are explained and how social phenomena are explained (Dennett^4^). This philosophical framework echoes in the cognitive systems theory that posits the existence of two potentially exclusive cognition systems, a social cognition system (i.e. processing of the social phenomena) and a physical cognition system (e.g., the processing of phenomena occurring in the non-social domain)^5–7^. This distinction has been supported by neural imaging studies providing evidence for two distinct neural networks that are specialized in processing information in one of these domains^6–8^. Interestingly, the social network (i.e. the network of brain regions that are involved in understanding and interacting with other people) shows overlap with the default mode network (i.e. a network of interacting brain regions that is active by default)^9^, leading some authors to propose that social cognition is the baseline/default state of thought^6,7^. Therefore, people might have the tendency to explain their environment in mentalistic terms by default, rather than entertaining more physical explanation. Thus, these strategies, or “stances”, explain and predict the behavior according to different levels of abstraction: 1—with reference to the *physical domain* of the agent, such as the trajectory of a ball (physical stance); 2—with reference to *how* the system was *designed* to function, for example, one expects the car to stop if they push the brake (design stance); 3—with reference to the agent’s *mental states and beliefs*, i.e. expecting that our friends would grasp a bottle of water when they say they are thirsty (intentional stance).

The social cognition system is obviously activated by observing other humans. However, apart from human conspecifics, also humanoid robots are one type of entities that might be capable of activating the social cognition system^10–12^, triggering mentalistic and social attributions^1,13,14^. In this context, it is important to examine how humans develop mental representation of robots. Mental representation are structured by processing external and internal information in working memory^15^. Here, we consider a general view of the working memory as a construct denoting a system that encodes, processes and retrieves stored and ongoing information for a limited amount of time^16^. Importantly, information processing in working memory is not without cognitive cost^17^.

By facing robots or any other agents, people develop a mental representation of the agent in working memory (encompassing physical or mental characteristics of the agent as well as contextual information) in a process that allows for making sense of their environments^15,18,19^. However, one feature that will determine whether the observers will incorporate more (or less) specific information related to the target (mechanistic information in the case of a robot) is the amount of cognitive resources available. Since we do not have an unlimited amount of cognitive resources, we need to select which information we will process or prioritize and how deep we will process it. The fewer available resources, the more superficially is the information processed. In other terms, we may consider that, under high cognitive load, people tend to use shortcuts to process information by using easily accessible information to build a representation. In HRI, the result would be using mentalistic (or anthropomorphic) representation to understand, and predict robots behaviours^1,20^.

However, the few recent studies investigating this issue show puzzling results. For instance, facing unpredictable behaviour people tend to attribute more intentionality to agents under cognitive load^21^. Conversely, Spunt and Lieberman^22^ showed that individuals tend to mentalize more when they are explicitly asked to. However, Spunt and Lieberman’s^22^ results showed that mentalization activity decreased as the function of the cognitive load when individuals are instructed to focus on the goals and intentions of the observed agent. To resolve these conflicting results we may refer to de Lange and colleagues study^23^. The authors showed that asking participants to reflect deliberately on goals and intentions could bias how the mirror neurons and mentalizing areas interconnect^23^ and could impact the synergy between the two systems^24,25^. In other words, explicit reflection on goals and intentions could bypass the default process of mentalization that is, according to Spunt and colleagues’ further study, automatically primed by the default mode network^26^. Therefore, we may assume that primed goal could produce different results than those obtained when cognitive load is manipulated and that focusing on goals and intentions would only result in bypassing the default mode, it is to say mentalizing agents’ actions.

## The present study

**Figure 1:**
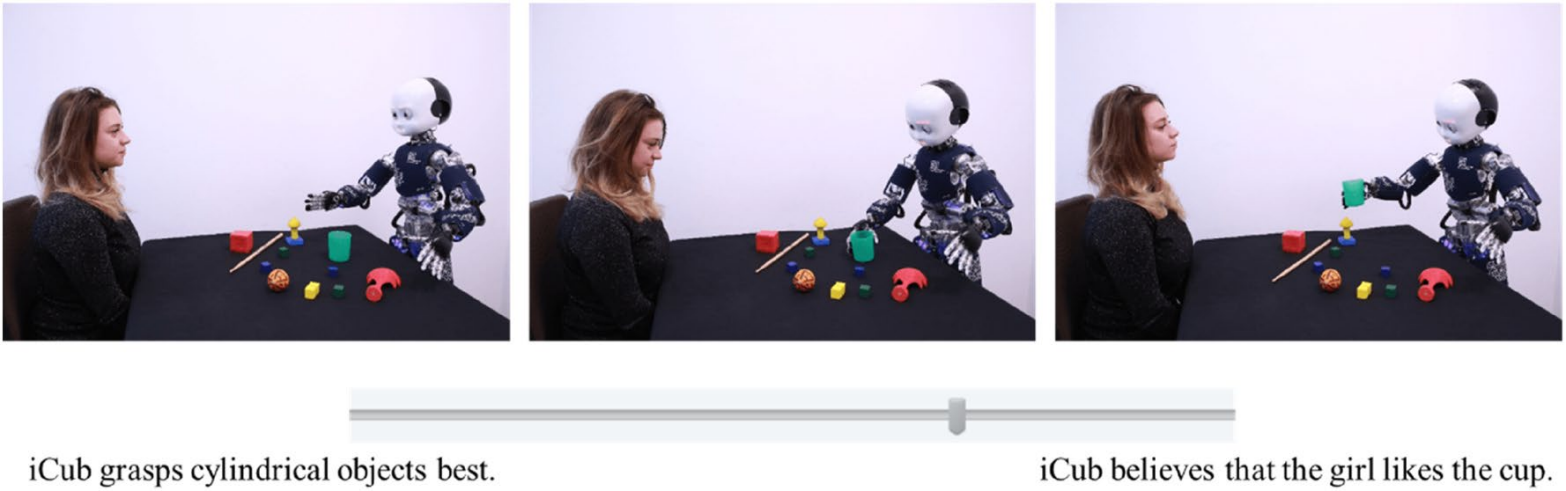
Example of an item from Marchesi et al.^27^.

In the present series of experiment, we sought to study the tendency to mentalize the behaviors of robots, taking into account the role of cognitive control and the depletion of cognitive resources during the observation. To do so, participants were asked to choose which description (mentalistic vs. mechanistic) fitted the best to scenarios depicting various robot actions^27^. The scenarios were designed by Marchesi et al.^27^ and depict an iCub robot (Metta et al.^28^) acting in various activities depicted in sequences of 3 pictures. Each scenario is both associated with a mentalistic and mechanistic description (Fig. 1). This paradigm allows a measure of attribution of intentionality without explicitly requiring participants to deliberate on the goal of the robot and has already been used in RT based experiments^29^.

We manipulated the level of cognitive load during the task (at various processing stages) to investigate the modulating role of cognitive resources on the adoption of intentional stance (i.e. mentalization bias). Assuming a two-phase model, in which at early stage information is accumulated with relatively limited selectivity to build the representation and a late stage which consists of using the representation (e.g. to formulate a judgment).

**Figure 2:**
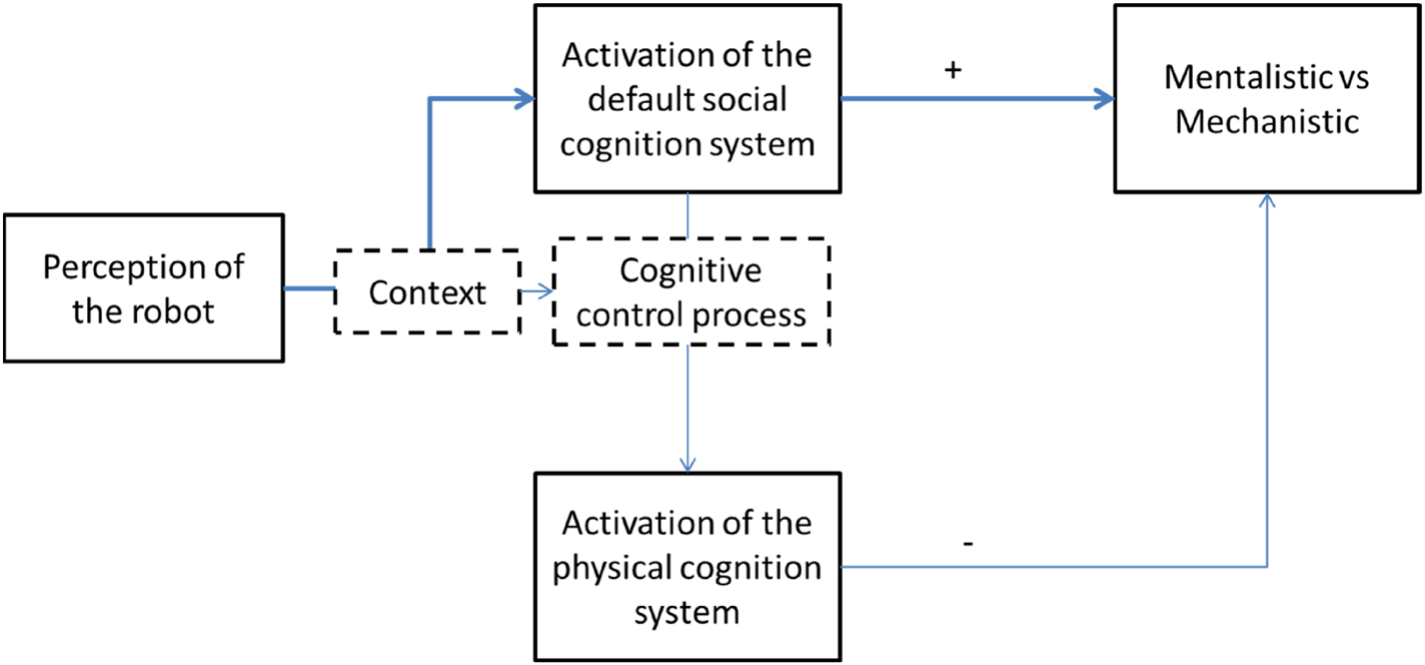
From the social and physical cognition system to mentalistic vs mechanistic representation of a robot’s actions.

We hypothesized that when individuals engage in explaining the behaviour of a robot under the situation of scarce resources, they will first develop a representation of the action using the most accessible representations at disposal. This strategy aims at reducing the cognitive cost while maintaining a control, an understandability of the situation. In addition, we have to consider that social cognition system (i.e. social-information processing of subjects) is more default than the cognitive domain related to physical systems (i.e. physical processing of objects)^9^. In other words, humans process social information of a scene by default (compared to physical information). Because the default mode network overlap with the social network^6,7,9,30,31^, the result would be, in an early stage, to form a representation of the robot’s behaviour with reference to mental states (i.e. mentalizing the behaviours, referring to beliefs, desires and intentions) faster than with reference to mechanistic states [43] as a form of automatic^32,33^ initial tendency stream^34,35^. However, because robots are not human, when a sufficient amount of cognitive resources are available, inhibition of the default path could occur and activate the physical cognition system instead^36,37^. With this system being active, mentalistic inferences stream would be bypassed and the weight of mechanistic information embedded in the representation of the scene should increase (Fig. 2). Importantly, while we consider mentalistic inferences as a default stream (faster than the alternative mechanistic one), we do not hypothesize uncontrollability^38,39^. In other terms, the higher speed of mentalistic inference (compared to mechanistic) is not controllable while the bypass, the ability to switch between the mentalistic and mechanistic stream is^33,40^. Therefore, the crucial question is not whether the switch occurs but *when* it occurs. As a consequence, in this series of study we are interested by the *speed rate* rather than *decision rate*.

In this study, we conducted 4 experiments to evaluate the impact of cognitive load on the *speed* to interpret robotic actions with mentalistic (compared to mechanistic) properties. We therefore measure the response time of participants (as a proxy for the mentalistic and mechanistic stream speed) to select between the two alternatives manipulating their level of cognitive load (high vs low) at different stage of the processing (early, when the mental representation is forming vs. late, when the judgment is elaborated).

## Experiment 1

The idea behind our study is that mentalistic and mechanistic descriptions result from the activation of the social and physical cognition systems, respectively.

The social cognition system is activated by default, the switch from social to physical processing should therefore result from a cognitive control process and, as a result, use cognitive resources. This reasoning entails that switching from a mental representation to a mechanistic one imposes a cognitive cost. Thus, the switch from a mentalistic representation of the scenario to a mechanistic one should be more difficult (slower) while there should be no difference between retaining a mechanistic representation, or switching from mechanistic to a mentalistic representation^41^. We tested this hypothesis by presenting mentalistic and mechanistic descriptions sequentially, asking participants to process and choose which description they thought best fits each scenario.

### Method experiment 1

#### Participants

Seventy-nine participants were recruited online to take part in this experiment (43 females, 30 males and 6 others, M_age_ = 22.5, SD = 4.9). All participants completed the experiment online in OpenLab^42^ and were not informed bout the purposes of the study The sample size was determined based on the desired power (0.80), alpha level (0.05), number of conditions (two in the main analysis)^43^, and anticipated medium effect size. Using G*Power 3.1^44^, the minimum required sample size was calculated as 66. As the experiment was conducted online, and all participants were recruited online on social media, we considered this minimum required sample size as a minimum per se, without setting a maximum threshold (the experiment remained accessible online for one week).

Before the beginning of the experiment, a screen described the data protection policies and participants’ rights in accordance with the European Union General Data Protection Regulation. This procedure was the same in all four experiments.

#### Procedure

**Figure 3:**
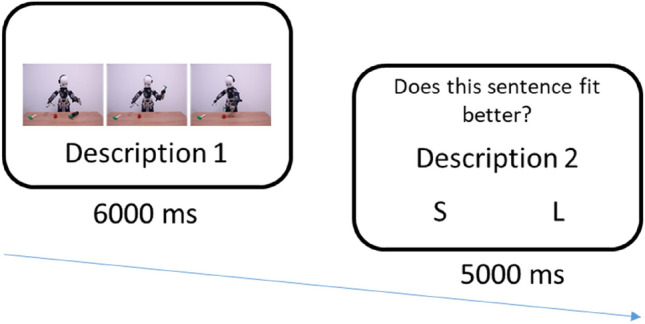
Experiment 1 trial sequence.

Participants were instructed that they will be evaluating scenarios depicting the action of a robot. These stimuli have already been used for RT measures^29^. For each trial (Fig. 3) a first description was presented with the scenario (6000 ms) and a second after the scenario (5000 ms). Participants had then to decide whether the second description described the scenario better than the first description using the S (“Change”) and L (“Retain”) keys. Before each trial, a 500 ms fixation cross was displayed at the centre of the screen. For half of the trials, the first description was mechanistic (e.g. iCub tracked the girl's hand movements”), for the other half, the first description was mentalistic (e.g. iCub understood that the girl wants the ball”). The order of items was randomly selected.

### Material experiment 1

To provide a sufficient number of trials to test the mentalistic-to-mechanistic (50% of the trials) and mechanistic-to-mentalistic switches (50% of the trials), we used the 34 items designed by Marchesi and colleagues^27^.

### Results experiment 1

#### Data pre-processing

We excluded response times (RTs) that were ± 3 standard deviations from participants’ individual mean per each individual condition. This resulted in exclusion of 1 trial that corresponded to 0.04% of trials. One participant was excluded from the analyses because constantly retained the first description.

#### Analyses

Analyses of Experiment 1 and the following experiments were conducted in R using the package lme4.

As recommended by Steegen and colleagues, and Botvinik-Nezer and colleagues^45,46^, we present two analyses (mixed model analysis on reaction times and linear integrated speed-accuracy scores) to evaluate the reliability of the results across statistical analysis choices. The second analyses makes it also possible to control for potential trade-off effects.

##### Response time analysis

To evaluate the RT of participants when changing or retaining the first description (mentalistic vs mechanistic) we conducted a mixed model analysis including the RTs of participants as dependent variable, the type of the first description (mentalistic vs. mechanistic) and the choice of the participant (change vs. retain) as within-participants factors. Also, we introduced the participants and the items as random factors.

**Figure 4:**
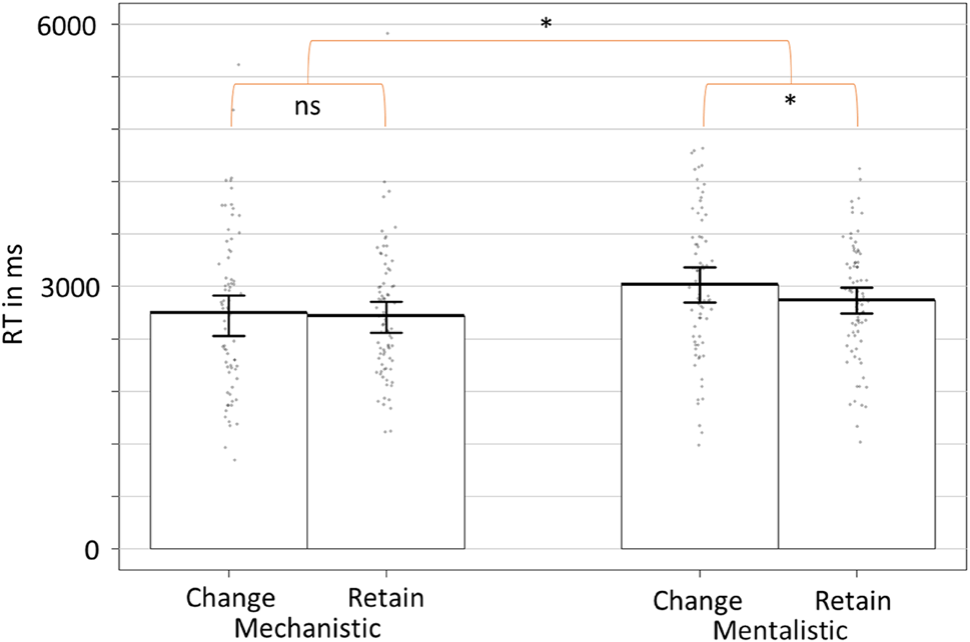
Time to choose retain/change between the first description and second description as a function of the description type (mechanistic vs mentalistic). * = *p* < 0.050.

Results showed an interaction between the type of the first description and the choice of participants, *B* = 177.54, *t*(2308.52) = 2.18, *p* = 0.034, *CI*_*95%*_ [12.90, 341.80] (Fig. 4). Contrast analyses with Bonferonni correction showed that while participants were faster when retaining the mentalistic description compared to changing to the mechanistic one, *B* = 155.20, *t*(1162.56) = 2.35, *p* = 0.019, *CI*_*95%*_ [24.93, 284.85], there was no significant difference when following the mechanistic description, *B* = − 53.08, *t*(1182.38) =  − 0.93, *p* = 0.408, *CI*_*95%*_ [− 178.98, 72.58]. In addition, as a main effect, results showed that participants were faster to change from mechanistic to mentalistic description compared to the opposite pattern, *B* = 373.79, *t*(46.26) = 3.90, *p* = 0.006, *CI*_*95%*_ [116.65, 626.09].

There was no difference on the proportion of choice after mentalistic or mechanistic description, *B* = − 0.01, *t*(75.14) = − 0.26, *p* = 0.799, *CI*_*95%*_ [− 0.10, 0.07].

#### Integrate score analysis

Analyses were also conducted on an adapted composite linear integrated speed-accuracy score (LISAS) (Vandierendonck^47,48^). The initial LISAS score represents response times weighted by the proportion of responses A vs an alternative response B. We adapted the computation to our paradigm and defined the new computed score (LISAS^b^) as RT_j_+ $\frac{Srt}{Spe}$  × PC_j_. RT_j_ is the participant’s mean RT in condition j, PC_j_ is the participant’s proportion of choice of response A (vs B) in condition j, S_RT_ is the participant’s overall standard deviation in RTs, and S_PE_ is the participant’s overall standard deviation in proportion of errors (PE). Weighting of the PE with the ratio of the RT and PE standard deviations is done to achieve a similar weight of the two components, RT and PE. This measure yields an estimate of RT corrected for the choice of participants (Vandierendonck^48^). This score allows to take into account both the response times of participants and the proportion of mentalistic vs mechanistic choices and to compute a score of “time to choose the mentalistic description weighted by the proportion of mentalistic vs mechanistic choices”.

In line with the mixed model, participants were faster to change from the mechanistic to the mentalistic description rather than the opposite, *F*(1, 77) = 5.11, *p* = 0.027, *CI*_*95%*_ [29.65, 468.29].

### Discussion experiment 1

Experiment 1 aimed to test whether the mentalistic representation is indeed a default. To do so, we tested whether the switch from a mentalistic representation to a mechanistic one was more effortful (in terms of cognitive resources demand) compared to the reverse.

The results showed that while there was no significant effect after the mechanistic description between retaining or changing, for a mentalistic alternative when the first description was mentalistic, participants were slower to change for the mechanistic alternative than retaining the mentalistic description. These results were confirmed by the LISAS analysis in which participants were faster to change from the mechanistic to the mentalistic description rather than the opposite.

Response times are a well-established method to evaluate the accessibility of information and the bias towards one representation compared to an alternative one^49,50^. Therefore, the present results argue for better accessibility of the mentalistic representation compared to the mechanistic one when describing a robot’s behaviour. Our change/retain paradigm makes it possible to confirm that it is more difficult to switch from a (default) mentalistic representation to a mechanistic representation than the opposite.

### Ethics

The study was approved by the local Ethical Committee (Comitato Etico Regione Liguria) and was conducted in accordance with the Code of Ethics of the World Medical Association (Declaration of Helsinki).

## Experiment 2

The second experiment aimed to test how the amount of cognitive resources available influences mentalistic versus mechanistic descriptions of robots’ behaviours when the two options are available at the same time. We hypothesized that, in high-cognitive load situation, participants should use accessible heuristics (i.e. mentalistic schemas) to interpret robot’s behaviours more easily (faster)^49,50^. Therefore, in high-cognitive-load compared to low-cognitive-load condition, in a binary choice between mentalistic and mechanistic descriptions of robot behaviours, participants should be faster to choose a mentalistic rather than a mechanistic option^18^.

### Method experiment 2

#### Participants

Seventy-two participants recruited online took part in this experiment (43 females, 24 males and 5 others, M_age_ = 21.2, SD = 4.4). All participants completed the experiment online on OpenLab and were not informed about the purpose of this study. For this experiment and the following one, the sample size was determined based on the desired power (0.80), alpha level (0.05), within design^43^, and anticipated medium effect size. Using G*Power 3.1^44^, the minimum required sample size was calculated as 66. Again we did not set a maximum threshold (the experiment remained accessible online for one week).

#### Procedure

Participants were instructed that they would be presented with various scenarios depicting a robot in daily activities. Their task would be to choose, among two descriptions, which one described best, according to them, the scenario depicted in the pictures using the S (left description) and L (right description) keys. One of the description involved mentalistic terms, while the other, mechanistic terms. In addition, participants were instructed that they had to remember a pattern matrix at the beginning of each trial. At the end of each trial, a second pattern matrix was displayed and they had to judge whether the two matrices were same or not. To respond, they used the S and L key of their keyboards. The purpose of introducing the matrices was to manipulate the amount of cognitive resources available for processing the presented scenario using complex (high-load) and simple (low-load) pattern matrices.

#### Task design

**Figure 5:**
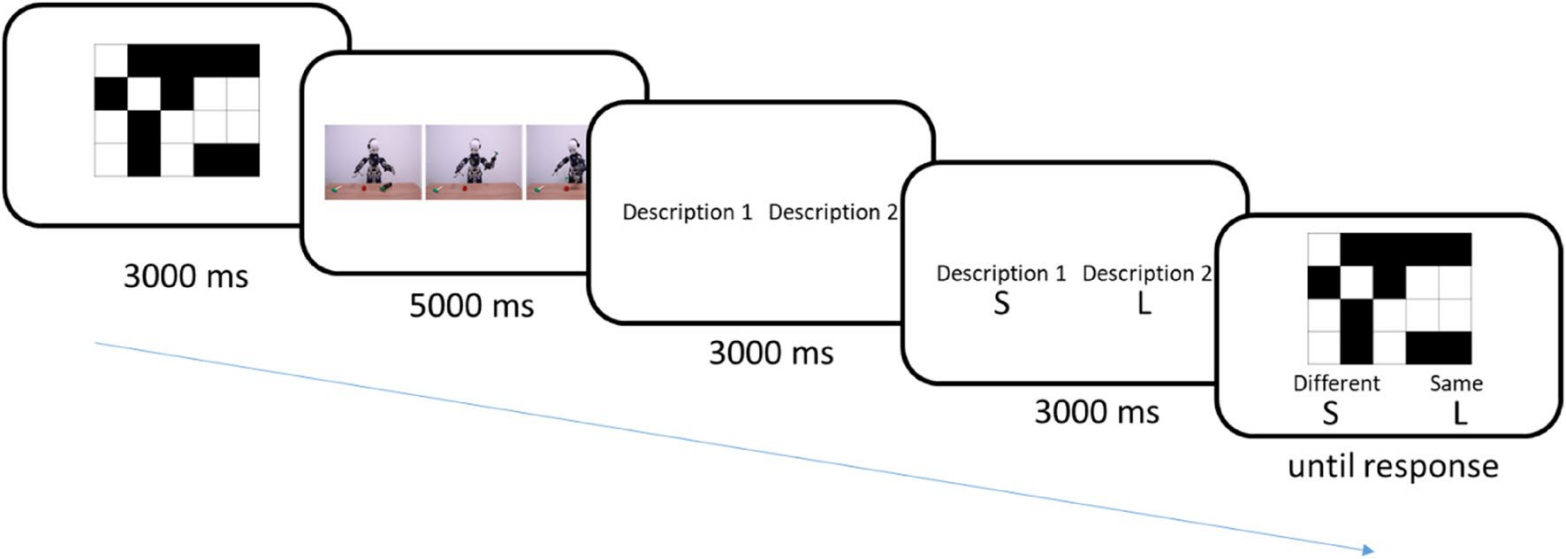
Experiment 2 trial sequence (without the fixation crosses).

The experimental design was as depicted in Fig. 5. First, each trial started with a fixation cross for 500 ms. Then, participants had to memorize, for half of the trials, a complex matrix, and for the other half, a simple matrix (3000 ms). Matrices were randomly selected (without replacement) for each trial. Complex matrices used 4 × 5 pattern with 10 black and 10 white squares. Simple matrices used 4 × 4 pattern matrices with 6 black and 6 white squares (The matrices were pretested with 20 participants, the average recall accuracy was 80.83% for the simple matrices and 70,83% for the complex matrices. The pretest consisted in the presentation of the matrices for 3000 ms, the presentation of 9 digits presented in a random order at the speed of 1 per second as a distraction, and the recall task without response time limit (*t*(19) = 3.79, *p* = 0.001, *CI*_*95%*_ [0.05, 0.16]).). A second fixation cross (500 ms) preceded the presentation of the scenario involving the robot. We used the scenarios designed by Marchesi et al.^27^ that depict an iCub robot (Metta et al.^28^) acting in various activities depicted in sequences of 3 pictures [5000 ms, the presentation time was pretested (Twenty participants were asked to evaluate between different presentation time (3000 ms, 4000 ms, 5000 ms, 6000 ms, 7000 ms), the minimum presentation time needed to accurately be able to describe the scenarios.)]. After the scenario, the two descriptions were presented. Participants were asked to respond to the scenario descriptions with “S” and “L” response keys, where S was mapped to the Description 1 (right) and L was mapped to Description 2 (left). The response keys were only activated after 3000 ms [defined by pretesting (Twenty participants were ask to evaluate between different presentation time (1000 ms, 2000 ms, 3000 ms, 4000 ms), the minimum presentation time of the sentences to be read. We choose the minimum presentation time to ensure control over the tendency to select the first sentence read and reduce intra-participant variability while not providing sufficient time to bias the hypothesized automatic process of mentalistic bias.)] to partially control the reading speed inter-individual differences. Finally, participants had to decide whether the new matrix displayed on the screen was similar to the first one with the “S” and “L” keys, where S was mapped to the NO response and L was mapped to the YES response for half of the trial, the other half displaying a reverse mapping to control for response carry-over effects. Half of the trials presented two identical matrices and the other half displayed different matrices.

#### Material

The experiment was programmed in JavaScript and displayed in participants’ web browser in full-screen using OpenLab^51^. The position of mentalistic/mechanistic description was counterbalanced (among the total 25 trials, 12–13 trials presented the mechanistic response on the right and 12–13 trials presented the mentalistic response on the right). We selected 25 scenarios among the 34 developed by Marchesi et al.^27^. Nine of the scenarios of the original set of Marchesi et al.^27^ were presented with descriptions that differed between mentalistic and mechanistic condition in more than 15 characters. This difference between mentalistic and mechanistic descriptions could bias the difficulty of processing the sentence and then bias participants' responses. This is why we excluded them from our present set of stimuli.

### Results experiment 2

#### Data preprocessing

Based on Cook’s distance we excluded two outliers^52,53^.

The RTs correspond to the time of response after the activation of the responses keys (or 3000 ms after the display of the descriptions). We then considered trials with reaction times (RT) lower or higher than 3 standard deviations from the mean per condition for each participant as outliers (for similar procedure see^54–57^. This criterion resulted in 0 trials excluded.

#### Analyses

##### Response times analysis

To evaluate the time of Mentalistic vs Mechanistic response selection, we conducted a mixed model analysis including the RTs of participants as dependent variable, the matrices’ difficulty (Complex vs Simple) and the choice of the participant (Mechanistic vs Mentalistic) as within-subjects factors. Finally, we introduced the participants and the items as random factors.

**Figure 6:**
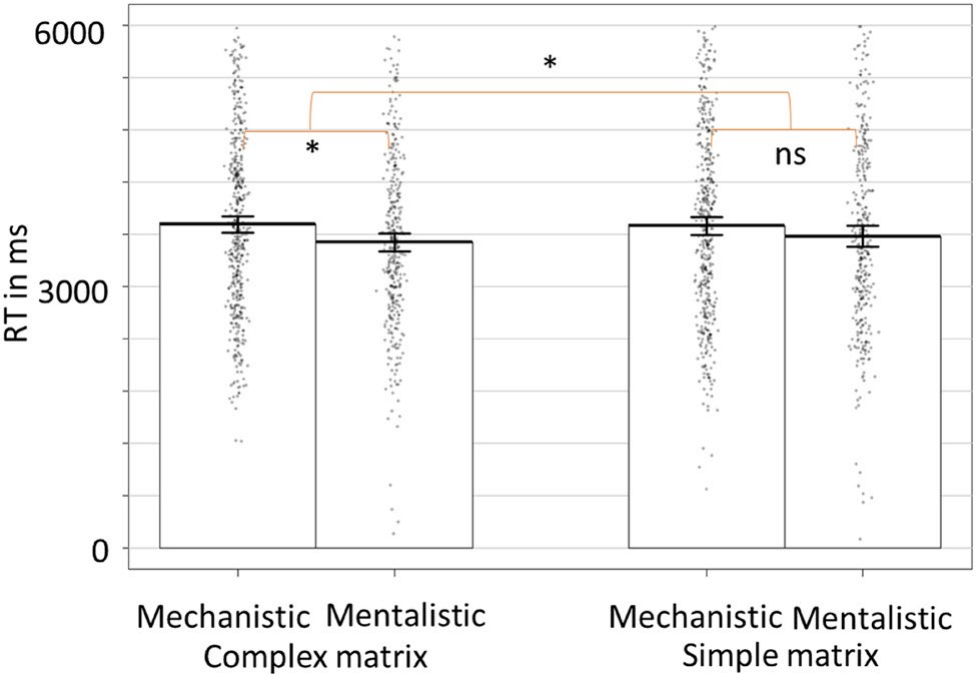
Time to select mechanistic and mentalistic descriptions as a function of the cognitive load level induced by the complex vs simple matrices. * = *p* < 0.050; ** = *p* < 0.010. The RTs correspond to the time of response after the activation of the responses keys (or 3000 ms after the display of the sentences).

The results showed an interaction of Matrix Difficulty by Choice on RTs, *B* = 181.18, *t*(1501.82) = 2.17, *p* = 0.031, *CI*_*95%*_ [17.09, 345.06] (Fig. 6). Contrasts with Bonferroni correction showed that, while there was no difference in simple matrix trials, *B* = − 69.23, *t*(765.36) =  − 0.96, *p* = 0.336, *CI*_*95%*_ [− 210.69, 71.74]; in complex matrix trials, participants were faster to select the mentalistic than the mechanistic explanation, *B* = − 207.22, *t*(784.51) =  − 3.18, *p* = 0.002, *CI*_*95%*_ [− 335.62, − 79.08]. We also found a main effect of Choice. Participants were faster to select the mentalistic response compared to the mechanistic one, *B* = − 221.87, *t*(1539.92) =  − 3.50, *p* = 0.001, *CI*_*95%*_ [− 346.19, − 97.65].

To check if the effects were not due to accuracy of matrices retrieval, we then isolated and examined only trials where participants accurately recalled (both complex vs simple) matrices. The interaction Difficulty by Choice was still significant *B* = 244.13, *t*(1022.62) = 2.40, *p* = 0.017, *CI*_*95%*_ [44.88, 443.19]. Analyses showed a main effect similar to the previous analysis, *B* = − 288.27, *t*(1061.54) =  − 3.57, *p* = 0.001, *CI*_*95%*_ [− 446.70, − 130.05].

Results did not show any significant difference in the number of mentalistic vs mechanistic choices in complex vs simple matrix trials, *B* = − 0.01, *t*(1517) =  − 15, *p* = 0.879, *CI*_*95%*_ [− 0.04, 0.04].

##### Integrated score analyses

We used the same procedure as presented in Experiment 1 to compute the integrated linear scores.

Including the time to choose the mentalistic response in complex vs simple matrix trials in a repeated measure ANOVA, we found an effect congruent with analysis on RT data. Participants took less time to choose the mentalistic description in complex compared to simple matrix trials, *F*(1, 69) = 4.82, *p* = 0.032, *CI*_*95%*_ [20.78, 434.38].

### Discussion experiment 2

The second experiment aimed to test whether the amount of available cognitive resources could influence participants’ time in choosing a description of robots’ behaviours using a mechanistic or a mentalistic vocabulary. While the analyses did not show any significant differences between mechanistic and mentalistic selection time in low cognitive load trials (simple matrices), when participants’ cognitive load was high (complex matrices), they were faster to select the mentalistic description of the robot’s behaviour compared to the mechanistic one. These results are in line with the idea that mentalization is a default mode of reasoning about others’ behaviour, which is also more accessible and cognitively less demanding.

However, the present results do not make it possible to disentangle whether the effect occurs at a late or early stage of processing. At a late stage, the cognitive load effect would occur during semantic processing of the mechanistic vs mentalistic descriptions content. According to this hypothesis, participants would be faster in choosing the mentalistic descriptions because mentalistic terms would be easier to process^58^. An alternative explanation could be that at an early stage, during the perception of the scene, the cognitive load could bias the mental representation of the scene in working memory. Considering that goal is encoded more strongly in memory and reactivated much more quickly than other more specific inferences^59^ and that mentalization descriptions are more related to goal than mechanistic descriptions ^3,60^, it would be easier for participants to rely on mentalistic descriptions to describe the scene^61^. Note that we do not assume these two interpretations as mutually exclusive.

## Experiment 3

In the third experiment, we investigated the late-stage cognitive load interpretation, while keeping the visual processing of the scene clear of any cognitive load manipulation. In this experiment, the perception of the scenario was the primary task and the memory task was only secondary. Participants could build a representation of the scenario before the cognitive load manipulation was introduced. The cognitive load occurred only during the choice between the mechanistic vs mentalistic descriptions. Therefore, the present experiment makes it possible to isolate the influence of the cognitive load on the later stage of processing when judgments are being made.

According to the late-stage interpretation, the cognitive load should impair the processing of the semantic content of the descriptions. As a result, the mentalistic terms relying on the default mode should be easier (faster) to use to describe the robot’s behaviours compared to the mechanistic terms. As such, we should observe lower response times for the mentalistic choices compared to mechanistic choices in high-load trials^62^.

### Method experiment 3

#### Participants

Seventy-two participants took part in this experiment on a voluntary basis (34 females, 25 males and 2 others, M_age_ = 20.8, SD = 3.6). All participants were recruited online and completed the experiment on OpenLab and were not informed about the purpose of this study.

#### Procedure

**Figure 7:**
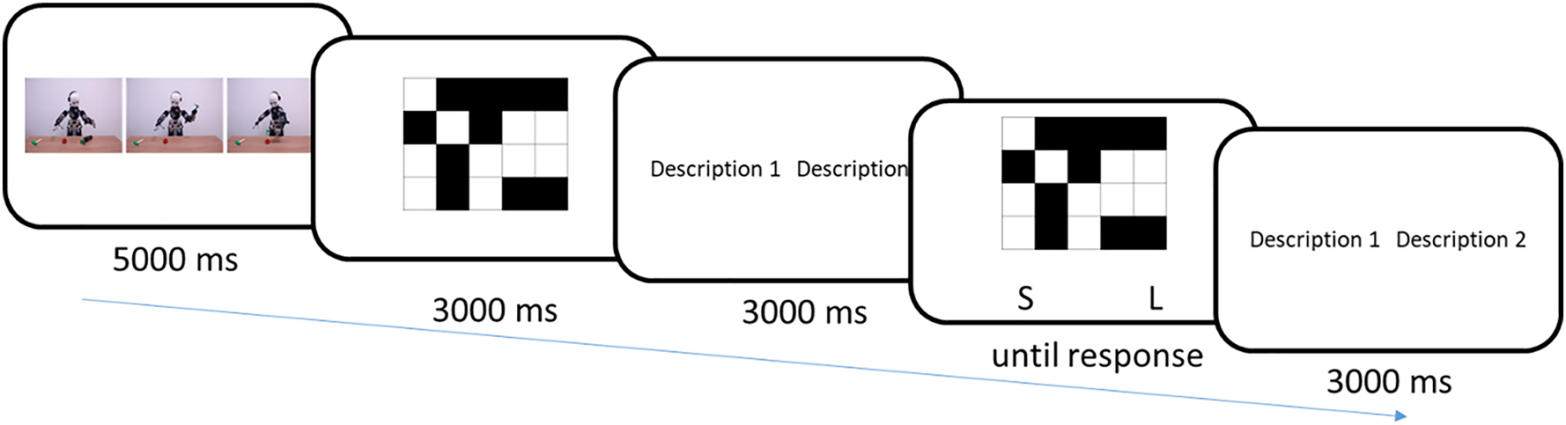
Experiment 3 trial sequence (without the fixation crosses).

The procedure was identical to the second experiment except that, in Experiment 3, the scenario was presented before the cognitive load manipulation (Fig. 7).

### Results experiment 3

#### Data preprocessing

The RTs correspond to the time of response after the activation of the responses keys (or 3000 ms after the display of the descriptions). Using Cook’s distance we excluded one outlier^52,53^. Correct trials with reaction times (RT) lower or higher than 3 standard deviations from the mean per condition for each participant were considered outliers and then removed from RT analyses, which corresponded to 4 trials (0.24% of trials).

#### Analyses

##### Response time analysis

To evaluate the time of Mentalistic vs Mechanistic response selection, we conducted a mixed model analysis including the RTs of participants as dependent variable, the matrix difficulty and the choice of the participant as within-subjects factors. Finally, we introduced the participants and the items as random factors.

Results did not show a significant interaction of matrix difficulty and choice, *B* = − 146.74, *t*(1581.76) =  − 1.30, *p* = 0.194, *CI*_*95%*_ [− 367.83, 74.35], no significant main effects (all p_s_ > 0.10). The same was true when controlling for only accurately recalled matrices (all p_s_ > 0.10).

Analyses on the frequencies of mechanistic vs mentalistic choices showed no difference in participants’ mechanistic/mentalistic descriptions choice in simple than complex matrix trials, *B* = − 0.02, *t*(1639.99) =  − 0.82, *p* = 0.410, *CI*_*95%*_ [− 0.07, 0.03].

##### Integrated score analysis

We used the same procedure as presented in Experiment 1–2 to compute the integrated linear scores. Including the time to choose the mentalistic response in complex vs. simple matrix trials in a repeated measure ANOVA, we did not find any significant effects (all *p*_s_ > 0.05).

### Discussion experiment 3

The third experiment aimed to test the effect of cognitive load on a late semantic processing stage during evaluation of robot behaviour in a mentalistic vs. mechanistic description decision task. Results showed no significant differences on response time and proportion of mentalistic choices in complex compared to simple matrix trials. In sum, and most importantly for the purposes of this study, results of Experiment 3 did not support the hypotheses that cognitive load affected late, semantic, stages of processing during evaluation of descriptions of robot behaviours.

## Experiment 4

Experiment 4 aimed to investigate the effect of the cognitive load on the stage of processing when mental representation of the presented scenarios is being built. If cognitive load affects this earlier stage of processing of the presented scenarios, participants should be faster to select a mentalistic description compared to a mechanistic one when the scenario was presented under a high cognitive load (complex matrix trials) compared to low cognitive load (simple matrix trials). The reason would be that goal representation would be encoded more strongly in memory and reactivated much more quickly than other more specific inferences^59^ favouring mentalistic descriptions of the scene^61^.

### Method experiment 4

#### Participants

Seventy-two participants took part in this experiment on a voluntary basis (32 females, 16 males, M_age_ = 20.7, SD = 6.1). All participants were recruited online and completed the experiment on OpenLab and were not informed about the purpose of this study.

#### Procedure

**Figure 8:**
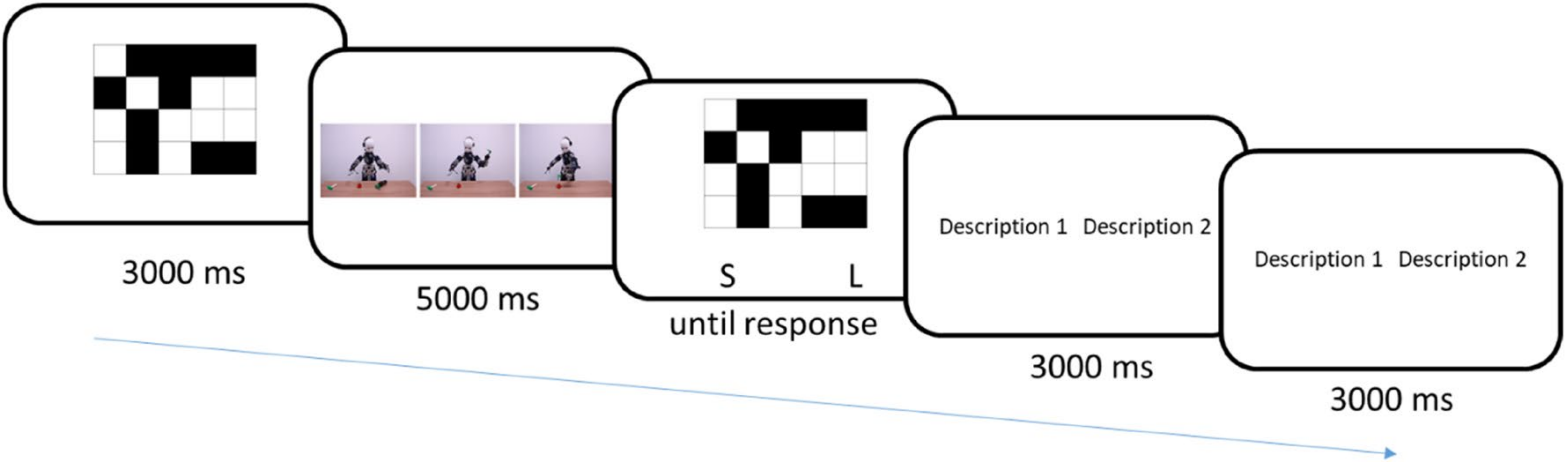
Experiment 4 trial sequence (without the fixation crosses).

The procedure was identical to Experiment 2 and 3 except that the cognitive load manipulation occurred before the scenario was presented and the recall occurred before the participants had to decide which description was the best fitting to the scenario (Fig. 8).

### Results experiment 4

#### Data preprocessing

The RTs correspond to the time of response after the activation of the responses keys (or 3000 ms after the display of the descriptions).As in previous experiments, we excluded response times (RTs) that were ± 3 standard deviations from participants’ individual mean, per each individual condition. This resulted in exclusion of 1 trial (0.06% of the trials).

##### Response time analysis

To evaluate the time of Mentalistic vs Mechanistic response selection as a function of the difficulty of the matrices, we conducted a mixed model analysis including the RTs of participants as dependent variable, the matrix difficulty and the choice of the participant as within-participants factors. Finally, we introduced the participants and the items as random factors.

**Figure 9:**
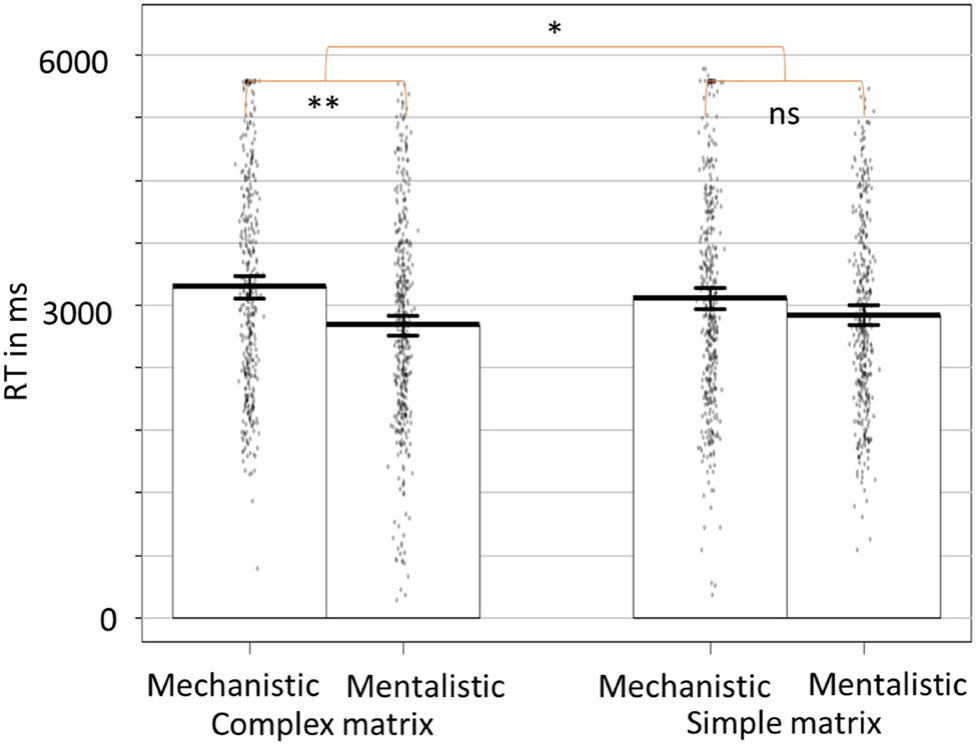
Time to select mechanistic or mentalistic descriptions as a function of the cognitive load induced by the complex vs simple matrices. The RTs correspond to the time of response after the activation of the responses keys (or 3000 ms after the display of the descriptions).

The results showed a significant interaction of difficulty and choice on RTs, *B* = 276.13, *t*(1486.16) = 2.87, *p* = 0.004, *CI*_*95%*_ [87.26, 464.93] (Fig. 9). Contrasts with Bonferroni correction showed that, while there was no difference in simple matrix trials, *B* = − 42.51, *t*(753.87) =  − 0.57, *p* = 0.568, *CI*_*95%*_ [− 189.69, 103.60]; in complex matrix trials, participants were faster to select the mentalistic compared to the mechanistic explanation, *B* = − 402.59, *t*(766.81) =  − 5.20, *p* < 0.001, *CI*_*95%*_ [− 554.48, − 251.03]. We also found a main effect of choice: participants were faster to select the mentalistic description compared to the mechanistic one, B = − 336.60, t(1512.88) =  − 4.74, p < 0.001, *CI*_*95%*_ [− 475.82, − 197.69].

We controlled for the effect of accuracy by isolating trials where participants accurately recalled the matrices. The interaction difficulty by choice (mentalistic vs mechanistic) reached significance, *B* = 337.27, *t*(679.82) = 2.47, *p* = 0.014, *CI*_*95%*_ [68.00, 606.27]. Analyses showed the same main effects; participants were faster to select the mentalistic description compared to the mechanistic one, *B* = − 278.79, *t*(697.03) =  − 2.78, *p* = 0.005, *CI*_*95%*_ [− 475.87, − 82.60].

Analyses on participants’ choice showed a higher number of mentalistic vs mechanistic choices in complex vs simple matrix trials, *B* = − 0.05, *t*(1506.81) = 2.01, *p* = 0.044, *CI*_*95%*_ [− 0.09, − 0.01].

##### Integrated score analysis

We used the same procedure as presented in Experiment 1–3 to compute the integrated linear scores. Including the time to choose the mentalistic response in complex vs simple matrix trials in a repeated measure ANOVA, unlike mixed model, the present result failed to reach significance (*p*_s_ > 0.05).

### Discussion experiment 4

Experiment 4 aimed to test the effect of cognitive load on speed of choosing mentalistic/mechanistic descriptions when the load was introduced during the processing of visual information depicting a robot’s actions. Our multiple-analysis approach yielded mixed effects. In response times, we found an impact of cognitive load on explanations (mentalistic vs. mechanistic). Participants were faster to select the mentalistic compared to the mechanistic response, but only in the high load condition. In the low load condition this effect was not observed, a pattern paralleling results of Experiment 2. However, the effect of load on speed of responding became non-significant in the second analysis on response times weighed by the proportion of mentalistic vs mechanistic choices.

To explain these mixed results we can formulate two hypotheses. First, a loss of statistical power switching from the mixed model to computed score analysis. Second, a cumulative effect when cognitive load impact both perception and judgment of the scenario. Indeed, the range of effect was higher in the Experiment 2 compared to Experiment 4. However, with respect to this second option, we have to consider that experiment 3, presenting cognitive load manipulation during judgment, did not show any significant results, therefore the cumulative effect hypotheses cannot be evaluated in details in the present series of experiments.

## General discussion

How humans explain robots’ action is a timely question with respect to the development of social robotics. Indeed, how people will behave towards robots, collaborate with them or accept them in their environment will depend on their representation of these artificial agents: will they be incorporated into the social cognition system? Or rather into a more “physical” system, related to non-social phenomena. The cognitive systems theory posits the existence of two potentially exclusive cognition systems: (i) a social cognition system, potentially the default system, and (ii) a physical cognition system^5–7^. While the social cognition system is default and more “accessible” (faster), the physical cognition system requires perhaps more effort. This has a direct implication for the relationship between the amount of available cognitive resources and processing information within each of the systems. Social cognition, being more accessible and default should require less cognitive resources than physical cognition. Thus, under high cognitive load, the more “physical”-cognitive processes should be affected more than socio-cognitive processes. This is what our study with four experiments aimed to test. Using the Instance items^27^ we evaluated to what extent (speed and decision) participants use mentalistic (vs mechanistic) information to describe the scenarios displaying robotic actions, as a function of high (vs low) cognitive load.

Experiment 1 was designed to first test the idea whether the mentalistic representation of robot actions (thus within the social cognition realm) is indeed default. We designed an experiment in which participants could either switch from one description of the scenario to another (from mentalistic to mechanistic or vice versa) or keep the initial description. If mentalistic representation is default, it should be more costly (i.e. slower) to switch from mentalistic to mechanistic than vice versa. This is indeed what our results showed.

In subsequent Experiments (2–4) we tested the idea that physical cognition representation should be more prone to interference by cognitive load than the more default, easier to access socio-cognitive reasoning. In Experiment 2 we introduced a cognitive load and we found that this was indeed the case. In the high cognitive load condition, participants were faster in choosing mentalistic descriptions relative to mechanistic descriptions. The remaining question was whether this interaction between cognitive load and social vs. physical reasoning processes occur at early or late stages of processing. In Experiment 3, we introduced the cognitive load at the stage of processing when participants should be evaluating the semantic content of the descriptions, leaving the earlier stages of processing when the mental representation is being formed untouched by the cognitive load manipulation. Results showed no effect of cognitive load on performance. However, when the load was introduced at an earlier stage of processing (Experiment 4), when the representation is being formed, it had a significant impact on the speed of choosing mentalistic vs. mechanistic descriptions, replicating results of Experiment 2 and arguing for a role of goal representation rather than purely linguistic differences.

In sum, our study showed that (i) interpreting behaviour of other (robotic) agents within the social cognition domain is more default (and less costly) than physical interpretations; (ii) social interpretation (social cognition)—being less costly—is easier (faster) to choose under high cognitive load condition, relative to mechanistic interpretation; (iii) the need for cognitive resources (and thus interference) occurs primarily at the earlier stages of processing, when representations are being formed.

To explain these results, we assume a cognitive control process that switches from the default mode of analysis that is the mentalization network to the more specific mode of analysis (in our case a mechanistic mode of analysis of robot’s behavior). This process would be modulated by the amount of cognitive resources available. As a consequence, when under cognitive load, people would rely on a default mode of thought (i.e., social cognition network), less demanding and more automatic^41,63–65^ because the control would be less effective. The result would be to analyze and build a mental representation of the scenes using more general and easy accessible information^66^. From a process perspective, it would be faster to accumulate evidence to fill a representation with (more general) mentalistic compared to (target specific) mechanistic information observing robotic actions. The control of this mentalistic bias would depend on the amount of resources available to bypass the social/mentalistic stream by a physical/mechanistic stream (Fig. 1). We may link this model to the anthropomorphism framework proposed by Epley and colleagues^1^. The dynamic would depend on the level of cognitive control modulated, for example, by the amount of cognitive resources available (as demonstrated in the present series of studies, but also perceptive features (e.g. human-likeness of the robot or the action^67^, or dispositional features (e.g. imaginative anthropomorphism^68^). Importantly, the timeframe for the effect of cognitive load (presented here) is early, during the generation of the representation. Therefore, we could summarize the process as follows: when perceiving a robotic agent, the observer develops a representation of this agent (and agent’s actions). During this phase, contextual and dispositional factors trigger the activation of the default social cognition system. The mentalistic inferences made based on this state may be controlled dependent on to the amount of resources available. In a subsequent phase of this dynamic process, once the representation is stable, the influence of contextual and dispositional factors decreases.

It remains to be discussed how long-term interaction affects this effect. Lemaignan and colleagues have proposed to distinguish three main phases in anthropomorphism: initialization, familiarization and stabilization^69^. During the initialization phase, anthropomorphism is proportional to the novelty effect^70^, the lack of knowledge to develop a target-specific representation about the robot^1^, the will to efficiently interact with the robot (and other motivational factors)^71^ and the activation of human-centric information^41^. The second phase is the familiarization phase when the user is getting acquainted with the robot. This phase corresponds to the development of the model of the robot using target-specific information rather than human-related information. A decrease in anthropomorphic attribution occurs at this phase because the initial apparent complexity of the robot diminishes. At last, the level of anthropomorphism in the stabilization phase is multi-factorial and will depend on the robot^72,73^, the user^71,73–77^ and the context^1,78^.

Therefore, considering this inevitable decrease in anthropomorphic attributions toward robots we could assume the same for mentalization. In line with this assumption is one of the most standard results in long-term HRI research: as the time goes by, the interest toward a robotic agent decreases^70,79–81^. The reason would be that when the novelty effect wears off, individuals lose interest and change their attitudes towards the artificial agent.

### Limitations of the study

One of the limitations of the present study is that we did not make a comparison between the robot to a human condition. It would be very interesting to compare mentalistic attribution across these two types of agents. However, it is very difficult to do so with our intentional stance tool, as it is difficult to provide mechanistic descriptions that make sense in the case of a human agent. Thus, due to the lack of this comparison, even if participants use more mentalistic inferences in high cognitive load condition for the human agent, we cannot discuss whether this phenomenon differs across the two types of agents.

Second potential limitation is that confidence intervals in the results of our study were relatively large, which argues for strong inter-individual differences. While we aimed to provide a general approach for the mentalization of robots in the cognitive control framework, it seems reasonable to assume that the present model is a simplified view of the process in which we could add dispositional but also cultural components^1^.

Third, we only used one type of robot. Research showed that the human-like appearance could influence the extent to which individuals attribute human-like characteristics to robots^82,83^. Therefore, manipulating the shape of the robot could reinforce or interfere with the weighting of social *vs.* physical processes. The nature of the relations between social and physical processes remains an open question. In the parallel-competitive model, both processes are activated in parallel streams that weigh automatic and controlled information to provide a single output^84^. In the default system theories, the social cognition system produces the initial output that can be corrected at a later stage by the physical cognition system, similar to evidence-accumulator models that are computed until the production of the final output^37^. Since the present results cannot argue in favour of one or the other model, future research could aim at disentangling the two theoretical proposals.

Fourth, participants may vary in their prior representation of robots which could potentially moderate the observed effect as well as personality traits such as the need for cognition or need for closure^73^. Therefore, in a follow-up study it would be relevant to investigate the effect of cognitive load on mentalization according to the prior representations of individuals.

### Future directions for examining cognitive load in HRI

Future studies could aim at disentangling the relationship between the physical cognition system and the default (social) system and how our social–cognition system processes input related to a humanoid robot^78^. Moreover, since our results suggest interindividual differences, future studies could explore and model the inter-individual variability in mentalistic attributions related to robots. Several factors, such as occupation or type of education can play a role in the inter-individual variability. One approach would be, for example, to model participants RTs with the help of Bayesian Inference or clustering algorithms. Such models allow researchers to take into account the inter-individual variability of participants during the task, and thus, allow for a better understanding of how the behaviour showed by participants changes along the experimental session, on a trial-by-trial basis. Finally, since robots are going to be more and more present in our daily interactions, it is crucial to understand how robust these results are across various contexts and how these results generalize to more daily-life scenarios. More specifically, it is crucial to examine which environmental and social context factors induce cognitive load in humans during human–robot interaction. One of clear candidate factor is certainly a learning context such as schools. Considering that, first, context may affect cognitive load; second, cognitive load the extent to which one would mentalize a robot; and third that mentalistic attributions to a robot may alter cognitive performance of individuals due to social presence effect induced by the robot^85,86^, or social comparison effects^87^, therefore it is crucial to understand clearly these interactions before introducing robots in schools.

## Conclusion

To better understand how humans engage in HRI, it appears inevitable to define the underlying cognitive mechanism involved. At first, people perceive and create representations of their artificial counterparts’ actions. This balance between representations and interpretations of these representations are poorly understood in HRI, which is paradoxical with respect to its social cognitive importance in social evaluation and interaction with both humans and robots^88,89^. The present series of experiments aimed at providing an understanding of the interaction between available cognitive resources and type of representation one builds. Our results show how that representing a robotic agent within a social cognition domain is more default and easily accessible, and thus occurs more readily when cognitive resources are less available as an interplay between a functional tendency and the environment. What follows is that representing a robot within the physical cognition domain is more effortful and thus less likely to be activated when cognitive resources are scarce. This pattern of results casts a light on how humans’ representation of a robots depends on their own cognitive state. In general, it shows that under cognitive load, humans tend to resort to the social cognition domain as an easily accessible mode of processing information. Activation of social cognition mechanisms is thus a shortcut for explaining behaviours of other systems, even if those systems are not humans, and could have important impact on how one could consider and behave toward these new artificial agents.

## Data Availability

All raw data will be available at OSF upon acceptance of the manuscript (https://osf.io/5gv7k/).
